# The yield of chest X-ray or ultra-low-dose chest-CT in emergency department patients suspected of pulmonary infection without respiratory symptoms or signs

**DOI:** 10.1007/s00330-023-09664-3

**Published:** 2023-04-28

**Authors:** Inge A. H. van den Berk, Emile H. Lejeune, Maadrika M. N. P. Kanglie, Tjitske S. R. van Engelen, Wouter de Monyé, Shandra Bipat, Patrick M. M. Bossuyt, Jaap Stoker, Jan M. Prins, on behalf of the OPTIMACT study group

**Affiliations:** 1 Department of Radiology and Nuclear Medicine, Amsterdam UMC Location University of Amsterdam, Meibergdreef 9, 1105 AZ Amsterdam, the Netherlands; 2 Department of Internal Medicine, Division of Infectious Diseases, Amsterdam UMC Location University of Amsterdam, Meibergdreef 9, Amsterdam, the Netherlands; 3 Department of Radiology, Spaarne Gasthuis, Boerhaavelaan 22, Haarlem, the Netherlands; 4 Department of Epidemiology & Data Science, Amsterdam UMC Location University of Amsterdam, Meibergdreef 9, Amsterdam, the Netherlands; 5 Amsterdam Public Health, Methodology, Amsterdam, The Netherlands; 6 Cancer Center Amsterdam, Imaging and Biomarkers, Meibergdreef 9, Amsterdam, the Netherlands

**Keywords:** Pneumonia, Tomography, X-ray computed, X-rays: thorax, Emergency service, hospital

## Abstract

**Objective:**

The yield of pulmonary imaging in patients with suspected infection but no respiratory symptoms or signs is probably limited, ultra-low-dose CT (ULDCT) is known to have a higher sensitivity than Chest X-ray (CXR). Our objective was to describe the yield of ULDCT and CXR in patients clinically suspected of infection, but without respiratory symptoms or signs, and to compare the diagnostic accuracy of ULDCT and CXR.

**Methods:**

In the OPTIMACT trial, patients suspected of non-traumatic pulmonary disease at the emergency department (ED) were randomly allocated to undergo CXR (1210 patients) or ULDCT (1208 patients). We identified 227 patients in the study group with fever, hypothermia, and/or elevated C-reactive protein (CRP) but no respiratory symptoms or signs, and estimated ULDCT and CXR sensitivity and specificity in detecting pneumonia. The final day-28 diagnosis served as the clinical reference standard.

**Results:**

In the ULDCT group, 14/116 (12%) received a final diagnosis of pneumonia, versus 8/111 (7%) in the CXR group. ULDCT sensitivity was significantly higher than that of CXR: 13/14 (93%) versus 4/8 (50%), a difference of 43% (95% CI: 6 to 80%). ULDCT specificity was 91/102 (89%) versus 97/103 (94%) for CXR, a difference of − 5% (95% CI: − 12 to 3%). PPV was 54% (13/24) for ULDCT versus 40% (4/10) for CXR, NPV 99% (91/92) versus 96% (97/101).

**Conclusion:**

Pneumonia can be present in ED patients without respiratory symptoms or signs who have a fever, hypothermia, and/or elevated CRP. ULDCT’s sensitivity is a significant advantage over CXR when pneumonia has to be excluded.

**Clinical relevance statement:**

Pulmonary imaging in patients with suspected infection but no respiratory symptoms or signs can result in the detection of clinically significant pneumonia. The increased sensitivity of ultra-low-dose chest CT compared to CXR is of added value in vulnerable and immunocompromised patients.

**Key Points:**

• *Clinical significant pneumonia does occur in patients who have a fever, low core body temperature, or elevated CRP without respiratory symptoms or signs*.

• *Pulmonary imaging should be considered in patients with unexplained symptoms or signs of infections*.

• *To exclude pneumonia in this patient group, ULDCT’s improved sensitivity is a significant advantage over CXR*.

**Supplementary Information:**

The online version contains supplementary material available at 10.1007/s00330-023-09664-3.

## Background

The classic clinical presentation of community-acquired pneumonia (CAP) is a febrile patient with respiratory signs and symptoms. In practice, however, the presentation of pneumonia varies. The symptoms and signs that can accompany pneumonia are non-specific, and no single symptom, sign, or combination thereof is a reliable predictor of the illness [1–3]. Some patients with pneumonia may not exhibit respiratory symptoms, others may not develop fever [3–5]. In particular elderly individuals and those with immunocompromising illnesses are less likely to develop fever [4, 6]. Therefore, radiographic criteria are often used for defining CAP [1, 3].

To date, chest X-ray (CXR) is the initial imaging modality of choice for detecting chest pathology. Although readily available, the diagnostic accuracy of CXR is limited [7–9]. Chest computed tomography (CT) is a three-dimensional projection technique without the disadvantage of over-projection, visualizing anatomy, and pathology better than CXR [10, 11]. With the advent of ultra-low-dose CT (ULDCT) the disadvantage of the higher radiation dose is largely overcome while diagnostic quality is preserved. In a systematic review and in a prospective study in the ED setting the sensitivity and specificity for diagnosing consolidations with ULDCT were 87–100% and 92–100%, respectively [12, 13]. Three prospective studies, one investigating patients in an outpatient clinic with ULDCT and the other two investigating patients in an emergency department (ED) setting with early chest CT, showed that (ULD)CT leads to an earlier diagnosis of pneumonia, which significantly affected clinical management and resulted in a significantly higher perceived confidence of the radiologist [8, 14, 15].

Pneumonia’s heterogeneous clinical presentation and, to some extent, the limited value of clinical diagnostic signs raise the question of whether pulmonary imaging should be considered in undifferentiated febrile patients who do not exhibit obvious respiratory symptoms. In four studies investigating either patients with neutropenic fever, with a fever of unknown origin, or febrile patients at the ED without respiratory symptoms, the yield of CXR varied between 2 and 5% [16–19]. The yield of pulmonary imaging in patients with a suspected infection but no respiratory symptoms or signs is therefore probably limited. It is currently unknown whether ULDCT also outperforms CXR in this group.

We recently reported the results of the OPTIMACT trial, a multicentre, non-inferiority, randomized clinical trial evaluating the health outcomes effect of replacing CXR by ULDCT in the diagnostic work-up of patients suspected of non-traumatic pulmonary disease at the ED [20]. The purpose of the sub-study reported here was to evaluate the yield of ULDCT and CXR in patients with a suspected infection who did not exhibit respiratory symptoms or signs, but in whom pulmonary infection needed to be ruled out. We hypothesized that ULDCT would result in a greater number of pneumonia diagnoses than CXR.

## Methods

### Study design

This study is an additional analysis of data collected in the OPTIMACT trial [20, 21]. In this trial, during randomly assigned periods of one calendar month between January 31, 2017, and May 31, 2018, either ULDCT or conventional CXR was used as the radiological imaging modality in patients who required pulmonary imaging at the ED of two participating Dutch hospitals: one university hospital (Amsterdam UMC) and one large teaching hospital (Spaarne Gasthuis (SG)) [21]. The Medical Ethics Committee of the Amsterdam UMC location AMC approved the study protocol. All participants provided written informed consent. The study was registered in the Netherlands Trial Register (number NTR6163).

### Study participants

The OPTIMACT study included patients 18 years and older, presenting at the ED suspected of non-traumatic pulmonary disease, and requiring chest imaging according to the attending physician. Patients could be self-referred or referred to the ED by their primary care physician or a hospital-based treating physician. Excluded were patients unable to undergo ULDCT or CXR, pregnant women, incapacitated patients, and patients with a life expectancy of less than one month or with other anticipated barriers to 28 days of follow-up data collection. Patients could only participate once [21]. Included in the present analysis were trial participants clinically suspected of infection but not presenting with respiratory signs or symptoms. Patients were suspected of infection when they had a fever (a core body temperature > 38.0 °C) or a low core body temperature (i.e., < 36.0 °C), or when they had an elevated (> 20 mg/L) C-reactive protein (CRP) level. Patients were not included in the analysis if they displayed at least one sign or symptom consistent with infectious respiratory disease, including coughing, sputum production, dyspnoea, hemoptysis, chest pain, or abnormal breathing sounds at auscultation.

### Study procedures

History taking, physical examination, and laboratory tests were initiated by the attending physician. After setting the indication for chest imaging and acquiring informed consent, the attending physician provided a clinical question on the structured and standardized radiology request form. This was followed by either ULDCT or CXR, according to the imaging method allocated to the month of presentation. If the clinical question was not adequately answered after obtaining the CXR or ULDCT, standard additional imaging (e.g., chest CT with intravenous contrast medium, CT pulmonary angiography) was performed. The median ULDCT dose was 0.2 mSv (IQR 0.2 to 0.3 mSv). The median CXR dose for portable anterior–posterior (AP) CXR was 0.02 mSv (IQR 0.02 to 0.03 mSv) and for bucky CXR posterior-anterior (PA) and lateral 0.05 mSv (IQR 0.03 to 0.07 mSv). Additional technical information on the imaging techniques used (ULDCT and CXR) is available in the Supplementary appendix.

Radiologists used a structured standardized report to optimize and standardize reading. Reading and reporting were performed or supervised by the radiologist on call at the time of clinical management, also outside office hours. The ULDCT and CXR were read with prior imaging if available. To increase inter-reader consistency, the residents and radiologists less experienced in the field of chest imaging were supervised by a group of seven radiologists with a subspecialty in chest imaging. When a radiologic diagnosis of pneumonia was made, a pneumonia pattern was reported: lobar, interstitial, or bronchopneumonia. Further details regarding the design of the OPTIMACT trial and the main outcomes are reported elsewhere [20, 21].

The data collected at baseline consisted of medical history, physical examination results, laboratory values, microbiological and radiological evaluation, and clinical diagnosis at ED discharge. Participants were followed for 28 days. All clinical, radiological, and microbiological data from participants available after 28 days of follow-up were reviewed post hoc. Based on these findings, a final day-28 diagnosis was assigned. A diagnostic handbook was developed to establish 32 possible day-28 diagnoses, including CAP, healthcare-associated pneumonia (HCAP), and aspiration pneumonia. Details of this handbook and its validation have been reported elsewhere [22]. All data were collected and stored in electronic case record forms (eCRF) (Castor EDC) [21].

Patients were categorized as immunocompromised if they had a history of human immunodeficiency virus (HIV) infection, organ or bone marrow transplantation, or hematological malignancy, had received recent chemotherapy (i.e., within the previous six months), were neutropenic, or if they used immunosuppressive medication (e.g., steroids or disease-modifying anti-rheumatic drugs (DMARDS).

### Statistical analysis

Between-group variations in categorical variables were compared with chi-squared tests or through Fisher’s exact test, as appropriate, continuous variables with unpaired *t*-tests or through Mann–Whitney U tests. We calculated the percentage of patients diagnosed with pneumonia by ULDCT or CXR. The day-28 diagnosis served as the clinical reference standard. In addition, we calculated and compared estimates of sensitivity and specificity for CXR and ULDCT in detecting pneumonia in this subgroup. All analyses were performed in IBM SPSS Statistics for Windows, version 26 (IBM Corp.).

## Results

### Study group

**Fig. 1:**
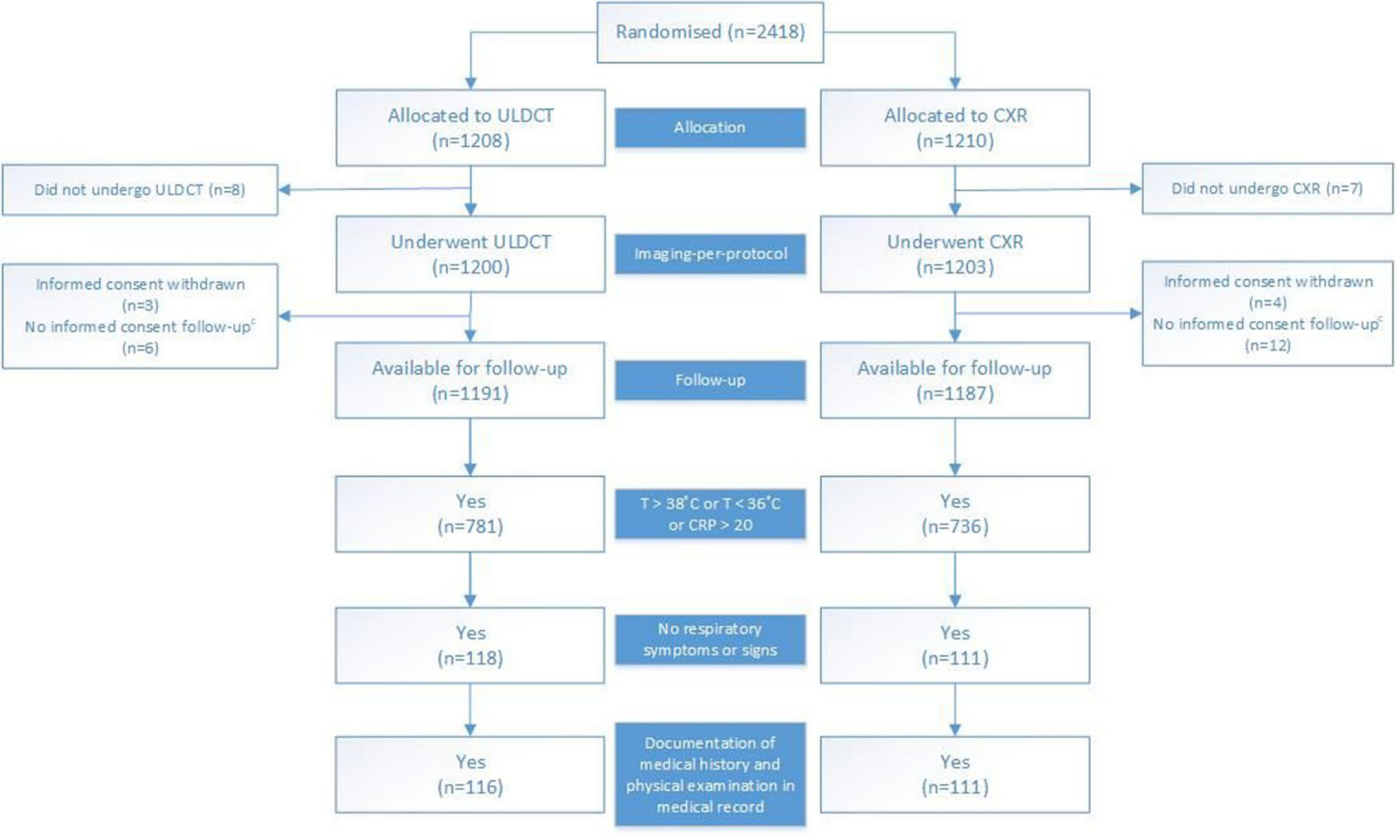
Flow chart. T, temperature; **°**C, Celsius; CRP, C-reactive protein; ULDCT, ultra-low-dose chest-CT; CXR, chest X-ray

Between January 31, 2017, and May 31, 2018, 2418 patients were included in the OPTIMACT study. Of these, 227 presented with fever, low core body temperature, or elevated CRP and no respiratory signs or symptoms; 116 were assigned to the ULDCT group and 111 to the CXR group (Fig. 1). The baseline characteristics of the ULDCT and CXR groups were broadly comparable (Table 1). In the ULDCT group, more patients had a hematologic malignancy, 21/116 (18%), versus 8/111 (7%) in the CXR group. In addition, more ULDCT patients had recently received chemotherapy, 28/116 (24%), versus 12/111 in the CXR group (11%), although the overall proportion of immunocompromised patients was comparable in both groups: 51/116 (44%) versus 39/111 (35%). There were no other significant differences in comorbidities, clinical parameters, or laboratory results. The clinical question on the radiology request form was most often (exclusion of) pneumonia: ULDCT 107/116 (92%), CXR 98/111 (88%).

**Table 1:** Baseline characteristics

Characteristics	ULDCT (*n* = 116)	CXR (*n* = 111)	*p*
Demographics			
Age in years, median [IQR]	59.0 [42–71]	57.0 [45–73]	0.3
Sex, male	56 (48)	49 (44)	0.5
Comorbidity			
Charlson Comorbidity Index (IQR)^a^	4 [2–5]	4 [1–6]	0.9
Immunocompromised	51 (44)	39 (35)	0.2
Recent chemotherapy	28 (24)	12 (11)	0.02
Solid tumor	23 (20)	24 (22)	0.7
Hematologic malignancy	21 (18)	8 (7)	0.02
Pulmonary disease			
COPD	4 (3)	5 (5)	0.7
Asthma	8 (7)	7 (6)	0.9
Cardiac disease			
Chronic heart failure	6 (5)	6 (5)	0.8
Myocardial infarction	12 (10)	11 (10)	0.9
Cerebrovascular disease	10 (9)	15 (14)	0.2
Diabetes	23 (20)	25 (23)	0.6
Renal disease	13 (11)	13 (12)	0.9
Liver disease	6 (5)	6 (5)	0.9
Symptoms			
Confusion	10 (9)	7 (6)	0.5
Clinical parameters and laboratory values			
Temperature, **°**C, mean [SD]	38 [1]	38 [1]	1.0
> 38 °C	81 (70)	69 (62)	0.3
< 36 °C	7 (6)	5 (5)	0.6
Heart rate, mean [*SD*]	96 [18]	95 [19]	0.4
Systolic blood pressure, mean [*SD*]	126 [21]	124 [23]	1.0
Respiratory rate, mean [*SD*]	18 [5]	18 [5]	0.7
Leucocytes, 10^**6**^/L, mean [*SD*]	9, 8 [6]	11, 1 [10]	0.4
Neutropenia, < 1.5 10^9^/L	2 (2)	1 (1)	1.0
CRP, mg/L, median [IQR]	40 [8–107]	41 [16–97]	0.7
CRP > 20 mg/L	75 (65)	78 (70)	0.4

Numbers are *n* (%), unless otherwise indicated. *ULDCT* ultra-low-dose chest computed tomography, *CXR* chest X-ray, *CRP* C-reactive protein, *COPD* chronic obstructive pulmonary disease, °C Celsius

^a^ Charlson Comorbidity Index, excluding AIDS. Predicts 10-year survival in patients with multiple comorbidities [23]

Pneumonia was categorized as CAP in 14 patients, as HCAP in 7 patients, and as aspiration pneumonia in one patient. More patients in the ULDCT group had a day-28 diagnosis of pneumonia: ULDCT 14/116 (12%) versus CXR 8/111 (7%), a difference of 5% (95% CI: 3% to 13%). The radiologist reported a lobar pneumonia pattern four times on ULDCT and two times on CXR, a bronchopneumonia pattern eight times on ULDCT and two times on CXR, and an interstitial pneumonia pattern was reported only once on ULDCT. The pneumonia pattern could not be specified in one respectively four cases.

The cases of pneumonia we found were clinically significant. At admission, the median CURB-65 score in patients diagnosed with pneumonia was 1 (IQR 0 to 2), and their median qSOFA-score (quick sequential organ failure assessment score) was 0 (IQR 0 to 1) [24, 25]. There were no patients that met all three qSOFA criteria. However, two patients presented with septic shock, and four patients required intravenous volume resuscitation because of hemodynamic instability. Most patients required admission and were prescribed antibiotics. Moreover, two patients that went undiagnosed by CXR at the ED later developed pneumonia visible on additional CXR and required admission.

When comparing patients with and without a final day-28 diagnosis of pneumonia (Table 2), we found no significant differences in demographics and comorbidities. More patients had a fever in the group diagnosed with pneumonia: 12/22 (55%), compared to 64/205 (31%) in patients with other diagnoses, a difference of 23% (95% CI: 2 to 45%). Median CRP was higher in patients with pneumonia: 69 (IQR 32 to 129) mg/L versus 35 (IQR 10 to 96) mg/L (*p* = 0.03). There was no significant difference in the number of patients with an elevated CRP (> 20 mg/L). No significant differences were found in the other clinical and vital parameters, the number of leukocytes, or the proportion of patients with neutropenia. There was no difference in admission rate between patients with pneumonia and those without: 15/22 (68%) versus 138/205 (67%). Patients diagnosed with pneumonia were more often treated with antibiotics: 19/22 (86%) versus 108/205 (53%), a difference of 33.7% (95% CI: 18 to 50%).

**Table 2:** Comparison between patients with or without a day-28 diagnosis of pneumonia

Characteristics	Pneumonia (*n* = 22)	No pneumonia (*n* = 205)	*p*
Demographics			
Age, mean [*SD*]	59.2 [17]	56.8 [19]	0.6
Male	10 (46)	95 (46)	1.0
Comorbidity			
Charlson Comorbidity Index (IQR)^a^	4 [2–6]	4 [1–6]	0.5
Immunocompromised	10 (46)	80 (39)	0.6
Recent chemotherapy	5 (23)	35 (17)	0.5
Solid tumor	6 (27)	41 (20)	0.4
Hematological malignancy	4 (18)	27 (13)	0.5
Pulmonary disease			
COPD	0 (0)	9 (4)	0.6
Asthma	2 (9)	13 (6)	0.6
Cardiac disease			
Chronic heart failure	0 (0)	11 (5)	0.6
Myocardial infarction	3 (14)	20 (10)	0.5
Cerebrovascular disease	4 (18)	21 (10)	0.3
Diabetes	5 (23)	43 (21)	0.8
Renal disease	3 (14)	23 (11)	0.7
Liver disease	0 (0)	12 (6)	0.6
Symptoms			
Confusion	2 (9)	15 (7)	0.7
Clinical parameters and laboratory values			
Temperature, ° C, mean [SD]	38.0 [1]	38.3 [1]	0.3
> 38° C	12 (55)	64 (31)	0.03
< 36 °C	1 (5)	11 (5)	1
Heart rate, mean [*SD*]	100 [21]	95 [18]	0.3
Systolic blood pressure, mean [*SD*]	123 [22]	126 [22]	0.6
Respiratory rate, mean [*SD*]	20 [5]	18 [5]	0.05
Leukocytes, 10^**6**^/L, mean [*SD*]	13 [10]	10 [8]	0.2
Neutropenia, < 1.5 10^9^/L	0 (0)	3 (2)	1
CRP, mg/L, median [IQR]	69 [32–129]	35 [10–96]	0.03
CRP > 20 mg/L	19 (86)	134 (65)	0.06
Outcome			
Hospital admission	15 (68)	138 (67)	1
Mortality within 30 days	1 (5)	4 (2)	0.4
Antibiotic treatment	19 (86)	108 (53)	0.002
Final day-28 diagnosis^b^			
Community-acquired pneumonia	14	0	
Healthcare-associated pneumonia	7	0	
Aspiration pneumonia	1	0	
Upper airway infection	0	7	
Fever of unknown origin	0	45	
Other thoracic pathology	0	9	
Pathology outside the chest	5	139	

Numbers are *n* (%), unless otherwise indicated. *ULDCT* ultra-low-dose chest computed tomography, *CXR* chest X-ray, *CRP* C-reactive protein, *COPD* chronic obstructive pulmonary disease, ^o^C Celsius

^a^Charlson Comorbidity Index, excluding AIDS. Predicts 10-year survival in patients with multiple comorbidities[23]

^b^ Day-28 diagnosis that occurred more than five times; a patient could have more than one diagnostic label

### Accuracy

When evaluating the diagnostic accuracy of ULDCT and CXR against the day-28 diagnosis, our clinical reference standard, we observed that 13/14 (93%) cases of pneumonia were correctly identified on ULDCT. Furthermore, 11/102 (11%) patients had a radiological diagnosis of pneumonia and no day-28 diagnosis of pneumonia (Table 3). In contrast, 4/8 (50%) cases of pneumonia were correctly identified by CXR but four others were missed; 6/103 (6%) patients had a radiological diagnosis of pneumonia and no day-28 diagnosis of pneumonia (Table 3).

**Table 3:** Cross-tabulation of radiological diagnosis and day-28 diagnosis for ULDCT and for CXR

	Day-28 diagnosis Pneumonia	Day-28 diagnosis No pneumonia	Total
ULDCT			
Pneumonia + on ULDCT	13	11	24
Pneumonia—on ULDCT	1	91	92
Total on ULDCT	14	102	116
Sensitivity 13/14 (93%) – Specificity 91/102 (89%) – PPV 13/24 (54%) – NPV 91/92 (99%)			
CXR			
Pneumonia + on CXR	4	6	10
Pneumonia − on CXR	4	97	101
Total on CXR	8	103	111
Sensitivity 4/8 (50%) – Specificity 97/103 (94%)– PPV 4/10 (40%) – NPV 97/101 (96%)			

*ULDCT* ultra-low-dose chest computed tomography; *CXR* chest X-ray; *PPV* positive predictive value; *NPV* negative predictive value

Therefore, ULDCT’s sensitivity for the detection of pneumonia was significantly higher (13/14, 93%) than that of CXR (4/8, 50%), a difference of 43% (95% CI: 6 to 80%). Specificity was 91/102 (89%) for ULDCT and 97/103 (94%) for CXR; the difference of − 5% (95% CI: − 12 to 3%) was not statistically significant. The positive predictive value of ULDCT was 13/24 (54%) and of CXR 4/10 (40%), a difference of 14% (95% CI: − 22 to 50%), the negative predictive value of ULDCT 91/92 (99%) and of CXR 97/101 (96%), a difference of 3% (95% CI: − 1 to 7%).

## Discussion

In this analysis of the OPTIMACT trial, we observed that pneumonia can occur in patients with fever or elevated CRP even if they do not show respiratory signs or symptoms. In total, 22 patients (10%) were diagnosed with pneumonia. ULDCT had a significantly higher sensitivity in the detection of pneumonia than CXR: 93% versus 50%. The cases of pneumonia we found were clinically significant, most patients required admission and were prescribed antibiotics. According to our findings, clinical characteristics or comorbidities alone cannot be used to identify patients with or without pneumonia, compatible with earlier observations that no single symptom, sign, or comorbidity is predictive of pneumonia [1–3], but on average more patients had a fever and CRP was higher in those diagnosed with pneumonia.

To our knowledge, only one other study has been done to investigate the yield of pulmonary imaging in ED patients who have a fever or elevated CRP without respiratory signs or symptoms [19]. This prospective cross-sectional study identified one single factor, a reliable history, that could discriminate between patients diagnosed with pneumonia and those who were not. The medical history was scored as reliable if the patient was not confused and had no language barrier or cognitive or verbal impairment. We did not see an association with pneumonia for any of the individual patient characteristics or clinical parameters that would construct a measure for reliable history. Most notably, no difference existed in the proportions of pneumonia and non-pneumonia in patients presenting with confusion. Interestingly, the proportion with pneumonia in our study group (10%; ULDCT 12%, CXR 7%) was relatively high compared with the overall proportion of 5% in the aforementioned cross-sectional study on this subject [19]. That trial relied on CXR to diagnose pneumonia. In our study, we relied on ULDCT or CXR. Our results clearly showed the higher sensitivity of ULDCT for the detection of pneumonia. The difference between both studies might also partly be explained by a different selection of subjects. The cross-sectional trial included patients from a regional hospital [19]. In our study, patients came from a university hospital and a regional teaching hospital. This can explain the notably high proportion of immunocompromised patients in our study group (40% versus 29%).

Compared to CXR, ULDCT had a significantly greater sensitivity for detecting pneumonia. A retrospective study in patients presenting with non-traumatic thoracic emergencies at the ED described CXR’s similarly low sensitivity (37.7%) for pneumonic consolidations when compared to conventional chest CT [7]. Another retrospective study reported 27% underdiagnoses of pneumonia in a series of 97 patients undergoing both CXR and conventional CT [10]. A recent systematic review and prospective study in an ED setting confirmed the high diagnostic accuracy of ULDCT, with a sensitivity for consolidations between 87 and 100% [12, 13]. Although the negative predictive value of both techniques is high (ULDCT 99%, CXR 96%), the very high sensitivity and very high negative predictive value of ULDCT is especially important when the prevalence of the disease is low and when in vulnerable, immunocompromised patients’ pneumonia has to be ruled out with certainty. This finding is in line with two prospective studies that suggested that the potential benefit of (ULD) chest CT lies in ruling out pneumonia and reducing the overdiagnosis of pneumonia [8, 15].

There were remarkably few patients with cardiovascular and pulmonary comorbidities in our group, which is likely due to the inclusion and exclusion criteria used. It is rare for patients afflicted with cardiovascular or pulmonary comorbidities to have no respiratory symptoms or signs [23]. Compared to the overall OPTIMACT study group, the patients in this analysis were more likely to have a history of malignancies, to have recently undergone chemotherapy, or to be immunocompromised. Even though these patients had no obvious respiratory signs or symptoms, the decision was made to perform pulmonary imaging. It is conceivable that, even when there are no apparent signs of respiratory disease, physicians are more likely to request pulmonary imaging in such patients.

Due to the pragmatic nature of the OPTIMACT trial, concealment of allocation was not possible. This may have influenced the behavior of physicians in including patients in the ULDCT or CXR months and may explain the higher proportion of patients with hematologic malignancies and recent chemotherapy in the ULDCT group. However, in our study group, we did not find a relationship between comorbidities and the presence of pneumonia. Second, due to the nature of this study, patients underwent either ULDCT or CXR, not both. Estimation of sensitivity and specificity was performed based on independent samples, while a more precise comparison would have been made in paired samples (i.e., CXR and ULDCT performed on the same patient). However, that would have prevented the evaluation of patient outcomes per diagnostic arm. Nonetheless, given the sample size, our results provide strong indications that ULDCT outperforms CXR in the detection of pneumonia in patients without respiratory signs or symptoms. The day-28 diagnosis was used as a reference standard, but results of the diagnostic strategy, ULDCT or CXR, were incorporated in the day-28 diagnosis, and this may have influenced the outcome. The influence is probably limited because the follow-up period of 28 days results in a post hoc day-28 diagnosis that uses all clinical, radiological, and microbiological data obtained during follow-up, including additional imaging and the natural course of the disease.

Finally, due to the small number of patients who were diagnosed with pneumonia, we had limited power to find patient characteristics that were indicative or predictive of pneumonia in patients without respiratory signs or symptoms.

Despite these limitations, this study’s strengths should also be highlighted. It was performed in the EDs of a tertiary and a large regional teaching hospital, where it included the complete spectrum of patients who present at the ED and who, according to the physician, require pulmonary imaging. This means that the results of this study are generalizable to other settings. Furthermore, this study was longitudinal in design, with a follow-up period of 28 days, and therefore the diagnosis of pneumonia could be established considering all clinical, radiological, and microbiological data from participants available after 28 days of follow-up. In this way, the clinical diagnosis of pneumonia could be established much more reliably than would have been possible when only the data from the moment of imaging would have been available.

In conclusion, our results imply that pulmonary imaging can at least be considered in patients who have a fever, low core body temperature, or elevated CRP without respiratory symptoms or signs. When the objective is to exclude pneumonia from the differential diagnosis in this patient group, for instance, because the patient is immunosuppressed, ULDCT’s improved sensitivity compared to CXR is a significant advantage.

### Supplementary Information

Below is the link to the electronic supplementary material.

Supplementary file1 (PDF 526 KB)

## Abbreviations

AMC: Amsterdam Medical Center
Amsterdam UMC: Amsterdam University Medical Center
CAP: Community-acquired pneumonia
CRP: C-reactive protein
CT: Computed tomography
CXR: Chest X-ray
DMARDS: Disease-modifying anti-rheumatic drugs
eCRF: Electronic case record forms
ED: Emergency department
HCAP: Healthcare-associated pneumonia
HIV: Human immunodeficiency virus
NTR: Netherlands trial register
OPTIMACT: OPTimal IMAging strategy in patients suspected of non-traumatic pulmonary disease at the ED: chest X-ray or CT
ULDCT: Ultra-low-dose computed tomography

## Declarations

### Guarantor

The scientific guarantor of this publication is Inge A.H. van den Berk.

### Statistics and biometry

One of the authors has significant statistical expertise.

No complex statistical methods were necessary for this paper.

### Study subjects or cohorts overlap

Some study subjects or cohorts have been previously reported in *Thorax*: van den Berk IAH, Kanglie M, van Engelen TSR et al. (2022) Ultra-low-dose CT versus chest X-ray for patients suspected of pulmonary disease at the emergency department: a multicentre randomised clinical trial. *Thorax*. https://doi.org/10.1136/thoraxjnl-2021–218337.

### Methodology

• prospective

• randomized controlled trial

• multicenter study

## References

[1] Gennis P, Gallagher J, Falvo C, Baker S, Than W (1989). Clinical criteria for the detection of pneumonia in adults: guidelines for ordering chest roentgenograms in the emergency department. J Emerg Med.

[2] Metlay JP, Kapoor WN, Fine MJ (1997). Does this patient have community-acquired pneumonia? Diagnosing pneumonia by history and physical examination. JAMA.

[3] Metlay JP, Waterer GW, Long AC (2019). Diagnosis and treatment of adults with community-acquired pneumonia. An Official Clinical Practice Guideline of the American Thoracic Society and Infectious Diseases Society of America. Am J Respir Crit Care Med.

[4] Musher DM, Thorner AR (2014). Community-acquired pneumonia. N Engl J Med.

[5] Moore M, Stuart B, Little P et al (2017) Predictors of pneumonia in lower respiratory tract infections: 3C prospective cough complication cohort study. Eur Respir J 50:1700434 10.1183/13993003.00434-2017 PMC5724402 29167296

[6] Mandell LA, Wunderink RG, Anzueto A (2007). Infectious Diseases Society of America/American Thoracic Society consensus guidelines on the management of community-acquired pneumonia in adults. Clin Infect Dis.

[7] Gezer NS, Balci P, Tuna KC, Akin IB, Baris MM, Oray NC (2017). Utility of chest CT after a chest X-ray in patients presenting to the ED with non-traumatic thoracic emergencies. Am J Emerg Med.

[8] Claessens YE, Debray MP, Tubach F (2015). Early chest computed tomography scan to assist diagnosis and guide treatment decision for suspected community-acquired pneumonia. Am J Respir Crit Care Med.

[9] Syrjala H, Broas M, Suramo I, Ojala A, Lahde S (1998). High-resolution computed tomography for the diagnosis of community-acquired pneumonia. Clin Infect Dis.

[10] Hayden GE, Wrenn KW (2009). Chest radiograph vs. computed tomography scan in the evaluation for pneumonia. J Emerg Med.

[11] Self WH, Courtney DM, McNaughton CD, Wunderink RG, Kline JA (2013) High discordance of chest x-ray and computed tomography for detection of pulmonary opacities in ED patients: implications for diagnosing pneumonia. Am J Emerg Med 31:401-405 10.1016/j.ajem.2012.08.041 PMC3556231 23083885

[12] Taekker M, Kristjansdottir B, Graumann O, Laursen CB, Pietersen PI (2021). Diagnostic accuracy of low-dose and ultra-low-dose CT in detection of chest pathology: a systematic review. Clin Imag.

[13] Taekker M, Kristjansdottir B, Andersen MB (2022). Diagnostic accuracy of ultra-low-dose chest computed tomography in an emergency department. Acta Radiol.

[14] Kroft LJM, van der Velden L, Giron IH, Roelofs JJH, de Roos A, Geleijns J (2019). Added value of ultra-low-dose computed tomography, dose equivalent to chest X-ray radiography, for diagnosing chest pathology. J Thorac Imaging.

[15] Prendki V, Scheffler M, Huttner B et al (2018) Low-dose computed tomography for the diagnosis of pneumonia in elderly patients: a prospective, interventional cohort study. Eur Respir J 51:1702375 10.1183/13993003.02375-2017 PMC5978575 29650558

[16] Navigante AH, Cerchietti LC, Costantini P (2002). Conventional chest radiography in the initial assessment of adult cancer patients with fever and neutropenia. Cancer Control.

[17] Oude Nijhuis CS, Gietema JA, Vellenga E (2003). Routine radiography does not have a role in the diagnostic evaluation of ambulatory adult febrile neutropenic cancer patients. Eur J Cancer.

[18] Bleeker-Rovers CP, Vos FJ, de Kleijn E (2007). A prospective multicenter study on fever of unknown origin: the yield of a structured diagnostic protocol. Medicine (Baltimore).

[19] de Stoppelaar SF, Pereverzeva L, Hafkamp B (2020). Diagnostic value of chest X-ray in patients with suspected infection and no respiratory signs or symptoms. Open Forum Infect Dis.

[20] van den Berk IAH, Kanglie MMNP, van Engelen TSR (2022). Ultra-low-dose CT versus chest X-ray for patients suspected of pulmonary disease at the emergency department: a multicentre randomised clinical trial. Thorax.

[21] van den Berk IAH, Kanglie MMNP, van Engelen TSR (2018). OPTimal IMAging strategy in patients suspected of non-traumatic pulmonary disease at the emergency department: chest X-ray or ultra-low-dose CT (OPTIMACT)-a randomised controlled trial chest X-ray or ultra-low-dose CT at the ED: design and rationale. Diagn Progn Res.

[22] van Engelen TSR, Kanglie MMNP, van den Berk IAH (2020). Classifying the diagnosis of study participants in clinical trials: a structured and efficient approach. Eur Radiol Exp.

[23] Labaki WW, Rosenberg SR (2020). Chronic obstructive pulmonary disease. Ann Intern Med.

[24] Singer M, Deutschman CS, Seymour CW (2016). The Third International Consensus Definitions for Sepsis and Septic Shock (Sepsis-3). JAMA.

[25] Lim WS, van der Eerden MM, Laing R (2003). Defining community acquired pneumonia severity on presentation to hospital: an international derivation and validation study. Thorax.

