# Supplementary material for: The yield of chest X-ray or ultra-low-dose chest-CT in emergency department patients suspected of pulmonary infection without respiratory symptoms or signs
Source: Eur Radiol. 2023 Apr 28;33(10):7294–302. doi: 10.1007/s00330-023-09664-3 (PMC10511555; doi:10.1007/s00330-023-09664-3)
Supplement: Supplementary file 1 — Supplementary file1 (PDF 526 KB) [file 330_2023_9664_MOESM1_ESM.pdf]

Supplementary Appendix

Van den Berk IAH, Lejeune EH, Kanglie MMNP et al. OPTIMACT study group. The yield of chest X-ray or ultra-low-dose Chest-CT in emergency department patients suspected of non-traumatic pulmonary infection without respiratory symptoms or signs.

Content

1. Members OPTIMACT study group
2. Technical information ULDCT and CXR

Members OPTIMACT study group

In alphabetical order, all affiliations are located in the Netherlands

Josje Altenburg, MD, PhD, Department of Pulmonology, Amsterdam UMC, University of Amsterdam, Amsterdam

Jouke Annema, MD, PhD, Professor of Pulmonary Endoscopy, Department of Pulmonology, Amsterdam UMC, University of Amsterdam, Amsterdam

Ludo F.M. Beenen, MD, Radiologist, Department of Radiology and Nuclear Medicine, Amsterdam UMC, University of Amsterdam, Amsterdam

Dominique Bekebrede-Kaufman, Radiology technician, Department of Radiology and Nuclear Medicine, Amsterdam UMC, University of Amsterdam, Amsterdam

Joost W. van den Berg, MD, Resident, Department of Internal Medicine, Amsterdam UMC, University of Amsterdam, Amsterdam

Inge A.H. van den Berk, MD, PhD candidate, Chest Radiologist, Department of Radiology and Nuclear Medicine, Amsterdam UMC, University of Amsterdam, Amsterdam

Sophie J. Bernelot Moens, MD, Resident, Department of Internal Medicine, Amsterdam UMC, University of Amsterdam, Amsterdam

Shandra Bipat, PhD, Clinical Epidemiologist, Department of Radiology and Nuclear Medicine, Amsterdam UMC, University of Amsterdam, Amsterdam

Bart G. Boerrigter, MD, PhD, Department of Pulmonology, Amsterdam UMC, Vrije Universiteit Amsterdam, Amsterdam

Marije M.K. Bomers, MD, PhD, Internist, Department of Internal Medicine, Amsterdam UMC, Vrije Universiteit Amsterdam, Amsterdam

Marjolein A.W. van den Boogert, MD, Resident, Department of Internal Medicine, Amsterdam UMC, University of Amsterdam, Amsterdam

Patrick M.M. Bossuyt, PhD, Professor of Clinical Epidemiology, Department of Clinical Epidemiology, Biostatistics and Bioinformatics, Amsterdam UMC, University of Amsterdam, Amsterdam

Merel L.J. Bouwman, MSc, Department of Internal Medicine, Center for Experimental and Molecular Medicine, Amsterdam UMC, University of Amsterdam, Amsterdam

Paul Bresser, MD, PhD, Pulmonologist, Department of Respiratory Medicine, OLVG, Amsterdam

Annemieke K. van den Broek, MD, PhD candidate, Department of Internal Medicine, Amsterdam UMC, University of Amsterdam, Amsterdam

Brenda Elzer, MSc, Research assistant, Department of Radiology and Nuclear Medicine, Amsterdam UMC, University of Amsterdam, Amsterdam

Marcel G.W. Dijkgraaf, PhD, Professor of Health Technology Assessment, Department of Clinical Epidemiology, Biostatistics and Bioinformatics, Amsterdam UMC, University of Amsterdam, Amsterdam

Jos Donkers, Patient Ambassador, Longfonds, Amersfoort

Elvin Eryigit, MD, Chest radiologist, Department of Radiology and Nuclear Medicine, Amsterdam UMC, Vrije Universiteit Amsterdam, Amsterdam

Tjitske S.R. van Engelen, MD, PhD candidate, Department of Internal Medicine, Center for Experimental and Molecular Medicine, Amsterdam UMC, University of Amsterdam, Amsterdam

Betty Frankemölle, Patient Ambassador, Longfonds, Amersfoort

Nina-Suzanne Groeneveld, BSc, Medical student, Faculty of Medicine, Amsterdam UMC, University of Amsterdam, Amsterdam

Maarten Groenink, MD, PhD, Cardiologist, Department of Cardiology, Amsterdam UMC, University of Amsterdam, Amsterdam

Emo E. van Halsema, MD, PhD, Resident, Department of Internal Medicine, Amsterdam UMC, University of Amsterdam, Amsterdam

Naomi M. Haverkamp Begemann, MD, Department of Radiology and Nuclear Medicine, Amsterdam UMC, University of Amsterdam, Amsterdam

Suzanne M.R. Höchheimer, MD, Emergency Physician, Emergency Department, Spaarne Gasthuis, Haarlem and Hoofddorp

David ten Hoff, BSc, Medical student, Faculty of Medicine, Amsterdam UMC, University of Amsterdam, Amsterdam

Frits Holleman, MD, PhD, MBA, Internist, Department of Internal Medicine, Amsterdam UMC, University of Amsterdam, Amsterdam

Erwin Hoolwerf, BSc, Medical student, Faculty of Medicine, Amsterdam UMC, University of Amsterdam, Amsterdam

Dorine Hulzebosch, Research nurse, Department of Radiology and Nuclear Medicine, Amsterdam UMC, University of Amsterdam, Amsterdam

Maadrika M.N.P. Kanglie, MD, PhD candidate, Department of Radiology and Nuclear Medicine, Amsterdam UMC, University of Amsterdam, Amsterdam and Department of Radiology, Spaarne Gasthuis, Haarlem and Hoofddorp

Mitran Keijzers, MD, Cardiologist, Department of Cardiology, Spaarne Gasthuis, Haarlem and Hoofddorp

Saskia Kolkman, MD, Radiologist, Department of Radiology and Nuclear Medicine, Amsterdam UMC, University of Amsterdam, Amsterdam

Jos A.J. Kooter, MD, PhD, Internist, Department of Internal Medicine, Amsterdam UMC, Vrije Universiteit Amsterdam, Amsterdam

Daniel A. Korevaar, MD, PhD, Resident, Department of Pulmonology, Amsterdam UMC, University of Amsterdam, Amsterdam

Ivo van der Lee, MD, PhD, Pulmonologist, Department of Pulmonology, Spaarne Gasthuis, Haarlem and Hoofddorp

Nick H.J. Lobe, Radiology technician, Department of Radiology and Nuclear Medicine, Amsterdam UMC, University of Amsterdam, Amsterdam

Peter A. Leenhouts, MD, MBA, Department of Emergency Care, Amsterdam UMC, University of Amsterdam, Amsterdam

Ramon B. van Loon, MD, PhD, Cardiologist, Department of Cardiology, Amsterdam UMC, Vrije Universiteit Amsterdam, Amsterdam

Paul Luijendijk, MD, PhD, Cardiologist, Department of Cardiology, Amsterdam UMC, Vrije Universiteit Amsterdam, Amsterdam

Melanie A. Monraats, MD, Radiologist, Department of Radiology and Nuclear Medicine, Amsterdam UMC, University of Amsterdam, Amsterdam

Bregje Mol, BSc, Medical student, Faculty of Medicine, Amsterdam UMC, University of Amsterdam, Amsterdam

Jan Luitse, MD, PhD, Department of Emergency Care, Department of Surgery, Amsterdam UMC, University of Amsterdam, Amsterdam

Lilian J. Meijboom, MD, PhD, Cardiothoracic Radiologist, Department of Radiology and Nuclear Medicine, Amsterdam UMC, Vrije Universiteit Amsterdam, Amsterdam

Carmen M. Melaan, MD, Faculty of Medicine, Amsterdam UMC, University of Amsterdam, Amsterdam

Saskia Middeldorp, MD, PhD, Professor of Medicine, Department of Vascular Medicine, Amsterdam Cardiovascular Sciences, Amsterdam UMC, University of Amsterdam, Amsterdam

Alexander Montauban van Swijndregt, MD, PhD, Chest Radiologist, Department of Radiology, OLVG, Amsterdam

Wouter de Monyé, MD, PhD, Radiologist, Department of Radiology, Spaarne Gasthuis, Haarlem and Hoofddorp

Jacqueline Otker, Bsc, LL.M., Patient Ambassador, Longfonds, Amersfoort

Jan M. Prins, MD, PhD, Professor of Medicine, Department of Internal Medicine, Division of Infectious Diseases, Amsterdam UMC, University of Amsterdam, Amsterdam

Anna Pijning, BSc, Medical student, Faculty of Medicine, Amsterdam UMC, University of Amsterdam, Amsterdam

Tom van der Poll, MD, PhD, Professor of Medicine, Department of Internal Medicine, Division of Infectious Diseases, Amsterdam UMC, University of Amsterdam, Amsterdam

Adrienne van Randen, MD, PhD ,Radiologist, Department of Radiology and Nuclear Medicine, Amsterdam UMC, University of Amsterdam, Amsterdam

Tom D.Y. Reijnders, MD, PhD candidate, Department of Internal Medicine, Amsterdam UMC, University of Amsterdam, Amsterdam

Milan L. Ridderikhof, MD, PhD, Emergency Physician, Department of Emergency Medicine, Amsterdam UMC, University of Amsterdam, Amsterdam

Johannes A. Romijn, MD, PhD, Professor of Medicine, Department of Internal Medicine, Division of Endocrinology, Amsterdam UMC, University of Amsterdam, Amsterdam

Jorien M. van Rooijen, MD, Resident, Department of Internal Medicine, Amsterdam UMC, University of Amsterdam, Amsterdam

Maeke J. Scheerder, MD, Radiologist, Department of Radiology and Nuclear Medicine, Amsterdam UMC, University of Amsterdam, Amsterdam

Antoinet J.N. Schoonderwoerd, Research assistant, Department of Radiology and Nuclear Medicine, Amsterdam UMC, University of Amsterdam, Amsterdam

Laura J. Schijf, MD, Radiologist, Department of Radiology and Nuclear Medicine, Amsterdam UMC, University of Amsterdam, Amsterdam

Frank F. Smithuis, MD, Radiologist, Department of Radiology and Nuclear Medicine, Amsterdam UMC, University of Amsterdam, Amsterdam

Ralf W. Sprengers, MD, PhD, EBCR, Cardiothoracic radiologist, Department of Radiology and Nuclear Medicine, Amsterdam UMC, Vrije Universiteit Amsterdam, Amsterdam

Robin Soetekouw, MD, Internist, Department of Internal Medicine, Spaarne Gasthuis, Haarlem and Hoofddorp

Jaap Stoker, MD, PhD, Professor of Radiology, Department of Radiology and Nuclear Medicine, Amsterdam UMC, University of Amsterdam, Amsterdam

Geert J. Streekstra, MSc, PhD, Department of Radiology and Nuclear Medicine and Department of Biomedical Engineering and Physics, Amsterdam UMC, University of Amsterdam, Amsterdam

Elizabeth M. Taal, MD, Research assistant, Department of Radiology and Nuclear Medicine, Amsterdam UMC, University of Amsterdam, Amsterdam

Milou M. Tjong Joe Wai, MD, Resident, Department of Radiology and Nuclear Medicine, Amsterdam UMC, University of Amsterdam, Amsterdam

Merve S. Tulek, BSc, Medical student, Faculty of Medicine, Amsterdam UMC, University of Amsterdam, Amsterdam

Glenn de Vries, BSc, Medical student, Faculty of Medicine, Amsterdam UMC, University of Amsterdam, Amsterdam

Daphne D.L. van der Velden, MD, PhD, Resident, Department of Radiology and Nuclear Medicine, Amsterdam UMC, University of Amsterdam, Amsterdam

Saskia Veldkamp, MD, Faculty of Medicine, Amsterdam UMC, University of Amsterdam, Amsterdam

Loek Verdegaal, BSc, Medical student, Faculty of Medicine, Amsterdam UMC, University of Amsterdam, Amsterdam

Maaike J.A. Vogel, Radiology technician, Department of Radiology and Nuclear Medicine, Amsterdam UMC, University of Amsterdam, Amsterdam

Lonneke A. van Vught, MD, PhD, Resident and postdoctoral researcher, Center for Experimental and Molecular Medicine (CEMM) and Department of General Medicine, Amsterdam UMC, University of Amsterdam, Amsterdam

Mart Vuurboom, MD, Faculty of Medicine, Amsterdam UMC, University of Amsterdam, Amsterdam

Guus A. Westerhof, MD, PhD, Resident, Department of Pulmonology, Amsterdam UMC, University of Amsterdam, Amsterdam

Pieta C. Wijsman, MD, Resident, Department of Pulmonology, Spaarne Gasthuis Haarlem and Hoofddorp

Michiel M. Winter, MD, PhD, Cardiologist, Department of Cardiology, Amsterdam UMC, University of Amsterdam, Amsterdam

Rosa D. Wouda, MD, PhD candidate, Department of Nephrology, Amsterdam UMC, University of Amsterdam, Amsterdam

Ibtisam Yahya, BSc, Medical student, Faculty of Medicine, Amsterdam UMC, University of Amsterdam, Amsterdam

Technical information ULDCT and CXR

At Amsterdam UMC ULDCT was performed at a Siemens Somatom Force (2015) with fixed 100kV, Sn filter, reference 50mAs, lung window slice reconstruction thickness 1.5 mm, filter Br54, Admire 3 was used. At SG ULDCT was performed at a Aquilion One Vision, Toshiba software 6.06ER0006 (2012, Canon Medical Systems) with fixed 120kV and 10mAs (20 mAs in adipose patients), lung window slice reconstruction thickness 2.0 mm, filter FC30, AIDR 3D strong. CXR was performed following standard procedures. At Amsterdam UMC CXR was performed on an Oldelft bucky at 125 kV (0.2mm Cu filter) using automated exposure control; at SG Siemens Ysio Max at 125 kV (0.2mm CU filter) using automated exposure control. CXR was performed posterior/anterior and lateral direction whenever possible. If not possible, anterior/posterior CXR will be performed using a mobile CXR device Carestream DRX Revolution in both participating centres.
